# Supplementary material for: The genome sequence of the Lesser Mountain Ringlet, Erebia melampus Füssly, 1775 (Lepidoptera: Nymphalidae)
Source: Wellcome Open Res. 2025 Jul 30;10:394. [Version 1] doi: 10.12688/wellcomeopenres.24659.1 (PMC12455180; doi:10.12688/wellcomeopenres.24659.1)
Supplement: Peer review history

# Reviewer report: Reviewer response for version 1

**DOI:** 10.21956/wellcomeopenres.27174.r131605

**Published:** 2025-09-22

**Authors:** Ricardo Rodriguez-de-la-vega

**Affiliations:** 1 CNRS, Université Paris-Saclay, Gif-sur-Yvette, France

In this Psyche project data note, the authors present a reference-standard, haplotype-resolved genome assembly of *Erabia melampus*, a brush-footed butterfly endemic to the Alps. As with the other genomes released by the project (77 as of September 20, 2025), the assembly combines high-quality HiFi reads with long-range Hi-C data. The hifiasm assemblies are excellent in terms of contiguity (the 424 Mb alternative haplotype assembled into just 164 scaffolds) and completeness (very few 31-mers exclusive to the reads).

 The presentation is well structured, the background offers a good primer to the species' ecology and the figures and tables adequately represent the underlying data. Readers are left with no explanation of what is the 8.4 Mb scaffold not assigned to arthropoda, but that's always the case in genome notes. I warmly welcome the inclusion of the Merian elements; even though there is understandably no room here for detailed discussion, it would have been interesting to place these findings in a comparative evolutionary genomics context.

 As far as I can tell, there's no RNAseq available yet. So I don't think the disclaimer "The genome will be ... at the European Bioinformatics Institute" in the "Data availability" section is justified here.

 I leave here the link to the GoaT link of the genome, as I think is a good entry point to access the numerous underlying data analysis.

 https://goat.genomehubs.org/record?recordId=1660630&result=taxon&taxonomy=ncbi#Erebia%20melampus

 I couldn't find a quality control page like the ones produced by the Darwin Tree of Life.

Are sufficient details of methods and materials provided to allow replication by others?

Yes

Is the rationale for creating the dataset(s) clearly described?

Yes

Are the datasets clearly presented in a useable and accessible format?

Yes

Are the protocols appropriate and is the work technically sound?

Yes

Reviewer Expertise:

Genomics, Bioinformatics, Evolution

I confirm that I have read this submission and believe that I have an appropriate level of expertise to confirm that it is of an acceptable scientific standard.

# Reviewer report: Reviewer response for version 1

**DOI:** 10.21956/wellcomeopenres.27174.r131608

**Published:** 2025-09-09

**Authors:** Laurence Despres

**Affiliations:** 1 Universite Savoie Mont Blanc, Chambéry, France; 2 Université Grenoble Alpes, Laboratory of Alpine Ecology (Ringgold ID: 56837), Grenoble, Auvergne-Rhône-Alpes, France

The authors report on the chromosome-level, haplotype-resolved genome sequence of a female specimen of the Lesser Mountain Ringlet *Erebia melampus* (Nymphalidae). The final assembly includes 21 chromosomes : 19 autosomes and both sex chromosomes Z and W. The mitogenome was also assembled. The quality of the assembly was further assessed by the proportion of complete BUSCO genes recovered, which is very high (>98% of Lepidoptera database BUSCO genes).

 The genome assembly is of good quality and was constructed using appropriate methods : Pac Bio HiFi long reads (29-fold coverage) and Hi-C data to confirm scaffolding, following the high standard of the Darwin Tree of Life Project for results presentation.

 Minor comment :

 A word (« diversified ») is missing in this sentence :

 ….its genome will provide invaluable opportunities to study genome evolution in one of the most karyological groups of butterflies ( Augustijnen *et al.*, 2024).

Are sufficient details of methods and materials provided to allow replication by others?

Yes

Is the rationale for creating the dataset(s) clearly described?

Yes

Are the datasets clearly presented in a useable and accessible format?

Yes

Are the protocols appropriate and is the work technically sound?

Yes

Reviewer Expertise:

evolutionary genomics

I confirm that I have read this submission and believe that I have an appropriate level of expertise to confirm that it is of an acceptable scientific standard.
